# Expression of CD22 in Triple-Negative Breast Cancer: A Novel Prognostic Biomarker and Potential Target for CAR Therapy

**DOI:** 10.3390/ijms24032152

**Published:** 2023-01-21

**Authors:** Tahir Zaib, Ke Cheng, Tingdang Liu, Ruyi Mei, Qin Liu, Xiaoling Zhou, Lifang He, Hibba Rashid, Qingdong Xie, Hanif Khan, Yien Xu, Pingnan Sun, Jundong Wu

**Affiliations:** 1 Stem Cell Research Center, Shantou University Medical College, Shantou 515041, China; 2 The Center for Reproductive Medicine, Shantou University Medical College, Shantou 515041, China; 3 Guangdong Provincial Key Laboratory of Infectious Diseases and Molecular Immunopathology, Shantou University Medical College, Shantou 515041, China; 4 The Breast Center, Cancer Hospital of Shantou University Medical College, Shantou 515041, China; 5 Guangdong Provincial Key Laboratory of Breast Cancer Diagnosis and Treatment, Shantou 515031, China; 6 Cancer Hospital of Shantou University Medical College, Shantou 515000, China; 7 Department of Health and Biological Sciences, Abasyn University, Peshawar 25000, Pakistan; 8 Department of Cell Systems and Anatomy, School of Medicine, University of Texas Health Science Center at San Antonio, San Antonio, TX 78229, USA

**Keywords:** triple negative breast cancer, CD22, CAR-T, biomarkers, immunohistochemistry

## Abstract

Triple-negative breast cancer (TNBC) accounts for 15–20% of all breast cancer cases. Due to the lack of expression of well-known molecular targets [estrogen receptor (ER), progesterone receptor (PR), and human epidermal growth factor receptor 2 (HER2)], there is a need for more alternative treatment approaches in TNBC. Chimeric antigen receptor (CAR)-T cell-based immunotherapy treatment is one of the latest treatment technologies with outstanding therapeutic advances in the past decade, especially in the treatment of hematologic malignancies, but the therapeutic effects of CAR-T cells against solid tumors have not yet shown significant clinical benefits. Identification of highly specific CAR-T targets in solid tumors is also crucial for its successful treatment. CD22 is reported to be a multifunctional receptor that is mainly expressed on the surface of mature B-cells (lymphocytes) and is also highly expressed in most B-cell malignancies. This study aimed to investigate the expression of CD22 in TNBC. Bioinformatic analysis was performed to evaluate the expression of CD22 in breast carcinoma and normal tissues. RNA-seq data of normal and breast carcinoma patients were downloaded from The Cancer Genome Atlas (TCGA), and differential gene expression was performed using R language. Additionally, online bioinformatics web tools (GEPIA and TNM plot) were used to evaluate the expression of CD22 in breast carcinoma and normal tissues. Western blot (WB) analysis and immunofluorescence (IF) were performed to characterize the expression of CD22 in TNBC cell lines. Immunohistochemical (IHC) staining was performed on tumor specimens from 97 TNBC patients for CD22 expression. Moreover, statistical analysis was performed to analyze the association of clinical pathological parameters with CD22 expression. Correlation analysis between overall survival data of TNBC patients and CD22 expression was also performed. Differential gene expression analysis of TCGA data revealed that CD22 is among the upregulated differentially expressed genes (DEGs) with high expression in breast cancer, as compared to normal breast tissues. WB and IF analysis revealed high expression of CD22 in TNBC cell lines. IHC results also showed that approximately 62.89% (61/97) of TNBC specimens were stained positive for CD22. Cell membrane expression of CD22 was evident in 23.71% (23/97) of TNBC specimens, and 39.18% (38/97) of TNBC specimens showed cytoplasmic/membrane expression, while 37.11% (36/97) specimens were negative for CD22. Furthermore, significant associations were found between the size of tumors in TNBC patients and CD22 expression, which unveils its potential as a prognostic biomarker. No significant correlation was found between the overall survival of TNBC patients and CD22 expression. In conclusion, we demonstrated for the first time that CD22 is highly expressed in TNBC. Based on our findings, we anticipated that CD22 could be used as a prognostic biomarker in TNBC, and it might be a potential CAR-T target in TNBC for whom few therapeutic options exist. However, more large-scale studies and clinical trials will ensure its potential usefulness as a CAR-T target in TNBC.

## 1. Introduction

Breast cancer, with a high occurrence rate of approximately 2.3 million cases per annum, is the most common malignant tumor worldwide [1]. The International Agency for Research on Cancer (IARC) published a report recently indicating that breast cancer cases are increasing across the globe each year. Breast cancer patients with early diagnoses always have a high chance of cure and survival, but the presence of heterogeneity in breast cancer tumors limits early diagnosis and targeted therapy [2]. Breast cancer is by far the most common malignancy in women, and younger women have less likelihood of getting breast cancer as compared to elderly women [3]. China accounts for approximately 12.2% of newly diagnosed breast cancer and 9.6% of all mortalities worldwide [4]. Breast cancer has now become the most frequently diagnosed cancer in Chinese women and the sixth most deadly cancer in China [5].

Identification of well-known biomarkers, e.g., estrogen receptor (ER), progesterone receptor (PR), and human epidermal growth factor receptor 2 (HER2), all of which are molecular targets of therapeutic agents, have achieved good outcomes in terms of survival and treatment of breast cancer patients. However, triple-negative breast cancer (TNBC), a heterogeneous subtype of breast cancer, lacks expression of ER, PR, and HER2 and accounts for approximately 15–20% of all breast cancer cases [6]. TNBC generally occurs in younger women, has a higher histologic grade, higher propensity to metastasize to distant visceral organs, high invasiveness, and worse outcome, with a high recurrence rate after adjuvant treatment, compared to other subtypes of breast cancer.

Due to the lack of specific targets, TNBC is not sensitive to endocrine therapy or current targeted therapy. Surgery, along with radiotherapy and chemotherapy, is currently the main treatment for patients with early-stage and advanced-stage TNBC, but its curative effect is unsatisfactory [7]. The effect of long-term chemotherapy is significantly reduced due to drug resistance, which easily results in tumor recurrence and distant metastasis [8]. The absence of crucial biomarkers in TNBC results in poor treatment of TNBC patients, but new immunotherapy treatment methods, such as Chimeric antigen receptor (CAR)-T cell and CAR-Natural killer (CAR-NK), have recently shown some encouraging results [9].

CAR-T cell immunotherapy has emerged as a promising immunotherapeutic strategy in TNBC [9]. This approach combines the antigen specificity of an antibody with the effector function of T cells. CAR-T cell-based immunotherapy treatment is one of the latest treatment technologies with remarkable therapeutic advances in the past decade, especially in the treatment of hematologic malignancies [10,11]. The basic concept of CAR-T cell technology is to genetically engineer T cells that recognize, and target tumor-specific antigens expressed on the surface of tumor cells to eradicate the tumor [12]. CAR-T technology has transformed the treatment of cancer to new heights in terms of overcoming the heterogeneity of cancer patients and provides an opportunity to cure more patients and improve survival rates.

Recognizing highly specific targets for CAR-T therapy against TNBC is critical for its effective treatment. Due to the complex and heterogenic nature of TNBC, there is always a need to explore new specific targets that could be used as antigens. CD22, a transmembrane glycoprotein and member of the immunoglobulin (Ig) superfamily, is reported to be a multifunctional receptor that is mainly expressed on the surface of mature B-cells (lymphocytes) and is also highly expressed in most B-cell malignancies [13]. The therapeutic advances targeting CD22 strongly recommend it as a CAR-T target. CAR-T cell therapy targeting CD22 in TNBC has not yet been investigated, and current studies lack evidence for the cell surface expression of CD22 in TNBC. In this study, we assessed the protein expression of CD22 by Immunohistochemical (IHC) staining in a large cohort of TNBC patients. We also performed statistical analysis to correlate the level and pattern of CD22 protein expression with different clinicopathologic parameters.

## 2. Results

### 2.1. Bioinformatics Analysis Reveals High Expression of CD22 in Breast Tumor

**Figure 1:**
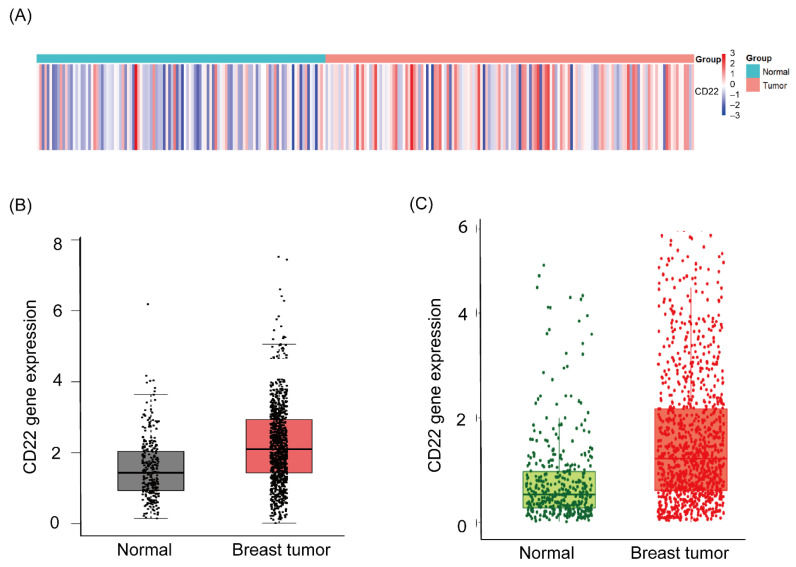
Bioinformatics analysis of CD22 expression in breast cancer and normal tissues. (**A**) Heatmap of the expression values for CD22 from RNA-seq data in TCGA. (**B**) BOX plot for CD22 expression from GEPIA shows that CD22 is relatively highly expressed in Breast tumor as compared to normal tissues. (**C**) TNMplot for CD22 expression also shows high expression of CD22 in breast tumor as compared to normal tissues.

Comprehensive analysis of RNA-seq data from TCGA data revealed that CD22 is among the upregulated, differentially expressed genes (DEGs) (LogFC >1). Heatmaps of CD22 gene expression clearly showed high expression of CD22 in most breast cancer tissue samples and that it reduced or had no expression in normal tissues (Figure 1A). When CD22 expression was investigated in breast cancer by Gene Expression Profiling Interactive Analysis (GEPIA) online database, the level of CD22 expression was slightly higher in breast cancer tissues than that in normal tissues (Figure 1B). TNMplot (a web application to enable a real-time comparison of gene expression changes between tumor, normal, and metastatic tissues amongst different types of platforms across all genes) analysis of CD22 expression in breast tumor and normal tissues also revealed that CD22 is relatively highly expressed in Breast tumor as compared to normal tissues (Figure 1C).

### 2.2. CD22 Is Highly Expressed in Breast Cancer Cell Lines

**Figure 2:**
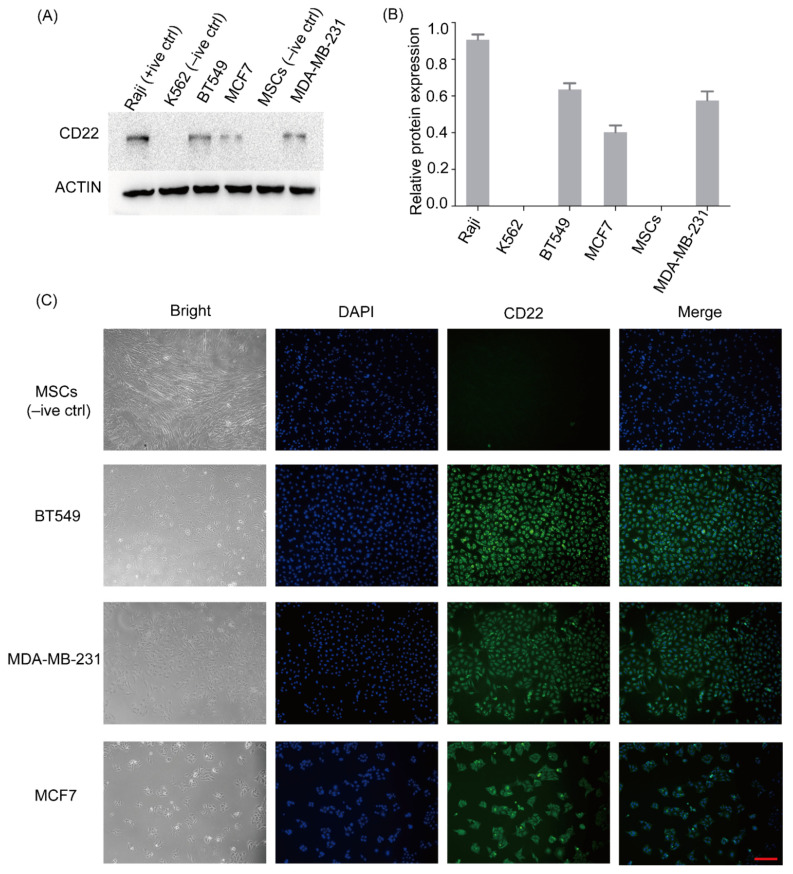
Expression of CD22 in breast cancer cell lines. (**A**) Western blot results for CD22 expression. BT549, MCF7, and MDA-MB-231 were positive for CD22 expression. Raji was used as the positive control, and K562 and MSCs were used as negative controls for CD22. (**B**) Graphical representation of relative CD22 protein expression values from the western blot analysis. (**C**) Representative images of CD22 expression by immunofluorescence of MSCs and breast cancer cell lines: BT549, MDA-MB-231, and MCF7. MSCs (negative control) showed no fluorescence. Membrane expression of CD22 stained in green can be seen in the BT549 and MDA-MB-231cell lines. Cytoplasmic expression of CD22 can be seen in the MCF7. Nuclei were stained with DAPI (4′, 6-diamidino-2-phenylindole) (blue). (Scale Bar: 100 µm).

To determine the expression of CD22 in breast cancer cell lines, western blotting (WB) was performed. WB analysis revealed that CD22 was expressed in BT549, MDA-MB-231, and MCF7 cells. Expression of CD22 is obvious in BT549. Raji cell line (positive control) expressed CD22, while K562 (negative control) and mesenchymal stem cells (MSCs) (negative control) did not show any expression for CD22. (Figure 2A,B).

### 2.3. Immunofluorescence Analysis Confirmed the Membrane Expression of CD22 in BT549 and MDA-MB-231 Breast Cancer Cell Lines

Immunofluorescence (IF) analysis of BT549 and MDA-MB-231 confirmed the membrane expression of CD22 in these cell lines (Figure 2C). Membrane expression of CD22 in these two TNBC cell lines indicates that CD22 can be a potential target for CAR-T treatment in TNBC. However, as a protein ectopic expressed glycoprotein, the glycosylation of CD22 in breast cancer cells may be different from B cells. More testing experiments, such as fluorescence-activated cell sorting, should be carried out in the future to identify the surface expression model of CD22 in breast cancer.

### 2.4. CD22 Is Highly Expressed in TNBC Specimens

**Figure 3:**
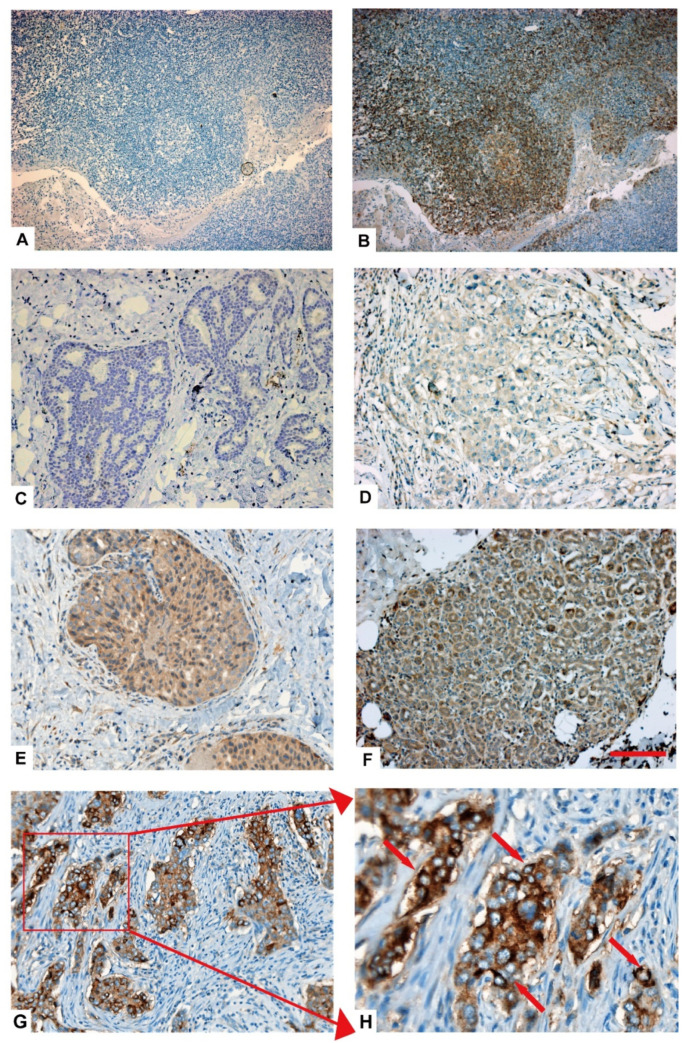
Representative images of immunohistochemical staining of CD22 expression in TNBC specimens. (**A**) Tonsillar carcinoma incubated with PBS showed no signal; (**B**) Tonsillar carcinoma incubated with anti-CD22 antibody showed positive expression for CD22; (**C**) TNBC sample negative for CD22. Relative expression of CD22 in TNBC samples (**D**) weak-positive, (**E**) moderate-positive, (**F**) strong-positive. (**G**,**H**) Membrane expression of CD22 in TNBC cells. The red arrows marked membrane expression of CD22 in TNBC cells. Magnifications: 20×, Scale bar 100 µm.

IHC staining of paraffin sections of 97 TNBC specimens revealed that CD22 is highly expressed in TNBC. Analysis of IHC results showed different levels of CD22 expression in TNBC tissues, i.e., negative, weak, moderate, and strong. Expression of CD22 protein in the cytoplasm and cell membrane of TNBC cells was evident in the IHC results (Figure 3). 

Tissue sample of tonsillar carcinoma was used as positive control of CD22 expression in IHC staining (Figure 3A,B). Different levels of cytoplasmic expression were observed, i.e., negative, weak-positive, moderate-positive, and strong-positive (Figure 3C–F). Overall, 62.89% (61/97) of TNBC specimens were positive for expression of CD22 (cytoplasmic and membranous), while the remaining 37.11% (36/97) of TNBC specimens were found to be negative.

Our IHC results showed that among the 97 TNBC specimens, 36 were negative, while 61 were positive for CD22 expression, among which 38 were cytoplasm-positive and 23 were cell membrane-positive (Table 1). Outcomes of our IHC results suggest that CD22 was widely expressed in TNBC. Furthermore, cell membrane expression of CD22 in 23 TNBC specimens suggests that CD22 can be utilized as a CAR-T target in TNBC.

### 2.5. Different Expression Levels of CD22 in TNBC Patient Tissues

Pathological scores were determined for CD22 expression in the tissue specimens of 97 TNBC patients (Table 2). A score of zero represented negative (Figure 3C), one represented weak-positive (Figure 3D), two indicated intermediate-positive (Figure 3E), and three indicated strong-positive (Figure 3F).

### 2.6. Association between Clinicopathological Features and Expression of CD22

We analyzed the association between the expression of CD22 and clinicopathological features (age, depth of tumor invasion, lymph node metastasis, histologic grade, clinical stage, and Ki67 expression) of 97 TNBC patients (Table 3, *p* < 0.05). Analysis revealed that CD22 expression was significantly associated with the size of the tumor (χ^2^ = 4.163, *p* = 0.041), while no significant associations were found with other clinicopathologic features, such as age (χ^2^ = 0.556, *p* = 0.456), lymph node metastasis (χ^2^ = 0.692, *p* = 0.707), histologic grade (χ^2^ = 1.740, *p* = 0.419), clinical stage (χ^2^ = 1.267, *p* = 0.260), and Ki67 expression (χ^2^ = 0.000, *p* = 1).

### 2.7. Correlation Analysis of Overall Survival of TNBC Patients and CD22 Expression

**Figure 4:**
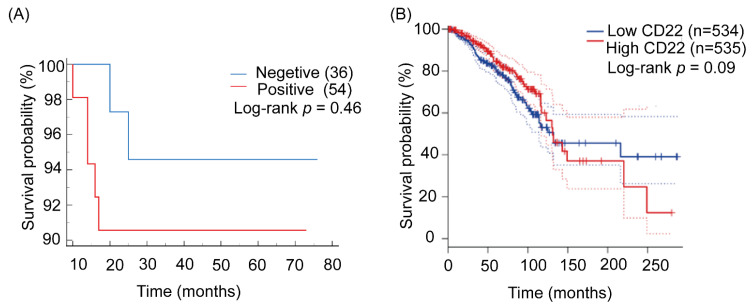
Correlation analysis of CD22 expression and overall Survival of TNBC patients (**A**) Correlation analysis of overall survival of TNBC patients and CD22 expression. (**B**) Survival plots for CD22 expression and breast cancer patients from GEPIA showed no significant association between both. The solid lines represent survival curve, and the dashed lines represent 95% confidence intervals.

Correlation analysis of overall survival data of TNBC patients and CD22 expression revealed worse overall survival for CD22-positive TNBC patients as compared to CD22-negative patients, but the difference was not statistically significant (*p* = 0.46) (Figure 4A). The low number of TNBC patients included in the study might be the reason for non-significance. Additionally, the survival plot for CD22 expression and overall survival of breast cancer patients from GEPIA was also not significant (*p* = 0.09) (Figure 4B).

## 3. Discussion

In this study, based on our bioinformatics analysis of TCGA data, we showed that CD22 was possibly expressed in TNBC cells and might be a novel and specific target for CAR-T cell therapy in TNBC, which currently lacks highly specific CAR-T targets. A comprehensive examination of breast cancer cell lines (BT549, MDA-MB-231, MCF7) for expression of CD22 by WB and IF staining, followed by IHC staining of 97 TNBC specimens, showed that CD22 is widely expressed in the cytoplasm and cell membrane of TNBC cells. As CD22 localizes to the cell surface, it might be a useful biomarker, especially in the diagnosis of advanced-stage cancer, and a potential target for CAR therapies in TNBC. Previously, CD22 expression has been linked with other malignancies, predominantly B-cell malignancies, where CAR-T therapy has shown success. Here, we report that CD22 is expressed in TNBC for the first time and propose it as a potential CAR-T target in TNBC.

Recently, CAR-T technology has achieved great success in the treatment of B-cell malignancies [14], but the success rate has not been so encouraging in the treatment of solid tumors. Multiple approaches have been recommended recently by scientists to enhance the efficacy and safety of CAR-T treatment in solid tumors, and selecting highly specific and optimal targets is also highly recommended [15]. Those specific targets could also play a crucial role in supporting clinical diagnosis, management, and treatment determination [16].

Ideal CAR-T targets are tumor-specific antigens (TSAs) that are only expressed in tumor cells. Although these antigens very rarely occur, they can contribute tremendously to the CAR-T treatment of cancer with less or no collateral damage to normal tissues. A high level of expression on tumor cells and being highly specific (expressed only on tumor cells) and highly stable (no loss of expression) are the qualities of an ideal CAR-T target that can assure the effectiveness of CAR-T treatment [15].

In CAR-T treatment of solid tumors, targets with high coverage or expression on cancer cells are very rare, and most of those targets currently reported for solid tumors have off-target effects that prevent treatment effectiveness [17]. Thus, the need for suitable targets with high tumor specificity is essential for avoiding or reducing toxicity necessary for successful CAR-T therapy. Mesothelin (MSLN) was reported to be the most specific immunotherapy target for TNBC until now [18].

For breast cancer treatment, several potential CAR-T targets are under investigation in clinical trials (http://clinicaltrials.gov/; accessed on 15 December 2021) (Table 4). However, the suitability of these antigens as CAR-T targets still needs to be proven in the future, as they are currently in clinical trials, while CD22 has already been shown to be a highly specific CAR-T target with great capacity and efficiency in clinical trials, especially in hematological malignancies. Many clinical trials have investigated the efficacy of immunotherapy targeting CD22 [19,20,21,22]. Thus, it is an appealing therapeutic target for autoimmune disorders and B-cell malignancies, and a variety of therapies targeting CD22 have already been developed, i.e., antibody–drug conjugates, CAR-T cells, etc. [23]. Comparing the protein expression values of CD22 with Mucin 1 (MUC1) (another favorable CAR-T target for TNBC) in normal human tissues using data from The Human Protein Atlas database (www.proteinatlas.org/; accessed on 15 December 2022) reveals that CD22 expressed in fewer normal tissues as compared to MUC1, which suggests that CD22 as CAR-T target in TNBC may result in less off-tumor toxicity. Hence, in the case of TNBC, which has very high heterogeneity, our finding that CD22 is expressed in TNBC cells can contribute greatly to the treatment of TNBC through CAR-T.

Some potential toxicity of CD22 as a CAR-T target is already known, and effective counter strategies have been developed to tackle it. For example, in the treatment of hematological malignancies, one of the main potential toxicities produced by CD22 as a CAR-T target is cytokine release syndrome (CRS) [24], and the drug tocilizumab (approved by FDA) is being extensively used to deal with CRS, which can block the activity of cytokines [25]. Scientists are continuously working on different strategies to reduce toxicity caused by CD22-CAR-T cell therapy [26].

Furthermore, the positive association between CD22 and the size of the tumor (one of the crucial clinical pathological parameters with considerable prognostic and predictive value) suggests that CD22 can be used as a prognostic biomarker in TNBC. Lately, the size of the tumor has been demonstrated as a crucial independent prognostic marker in breast cancer, especially in patients with extensive nodal status [27]. Some other studies on colon and esophageal cancer also emphasized the importance of the size of the tumor as a crucial clinical pathological parameter and recommended its incorporation into the staging system to facilitate the prediction of death and recurrence risk [28,29]. Additionally, in our survival analysis of the results for 90 TNBC patients, we saw worse overall survival for CD22-positive TNBC patients as compared to CD22-negative patients, but the survival difference was not statistically significant (*p* = 0.46) (Figure 4A). Therefore, the correlation between CD22 expression and the overall survival of TNBC patients remains uncertain.

CD22-associated signaling pathway in B cells involves Src homology region 2 domain-containing phosphatase 1 (SHP-1), which inhibits B cell receptor signaling upon attachment with phosphorylated ITIMs (immunoreceptor tyrosine-based inhibitory motifs) of CD22 [30]. Recently, Ballet et al. demonstrated that B cells with reduced SHP-1 activity or expressing CD22 with mutated SHP-1 bindings altered the normal functioning of B cells [31]. Interestingly, SHP-1 has also been reported to act as a tumor suppressor in breast cancer [32], and its expression is negatively correlated with EGFR (Epidermal growth factor receptor) [33], which is highly expressed in 15–45% of breast cancer [34]. In our study, high expression of CD22 in TNBC might also be negatively correlated with SHP-1, and CD22 might contribute to TNBC tumor progression.

In conclusion, we demonstrated that CD22 is highly expressed in TNBC. Based on our findings, we anticipated that CD22 could be a potential CAR-T target in TNBC for which few therapeutic options exist. However, more large-scale studies and clinical trials will ensure its potential usefulness as a CAR-T target in TNBC.

## 4. Materials and Methods

### 4.1. Collection of TNBC Tumor Samples

We obtained 97 formalin-fixed and paraffin-embedded TNBC samples from the archives of the Cancer Hospital of Shantou University Medical College, Shantou, China. These 97 TNBC patients underwent primary radical surgical resection from 2016 to 2020 in the Cancer Hospital of Shantou University Medical College, Shantou, China. These patients had not received chemotherapy and radiotherapy before surgery. This study was approved by the Ethics Committee of the Affiliated Cancer Hospital of Shantou University Medical College (protocol no. 2022153).

### 4.2. Bioinformatics Analysis

#### CD22 Expression in Normal and Breast Cancer Tissues Was Analyzed Using Public Databases

To analyze whether CD22 is highly expressed in breast cancer, differential gene expression analysis was performed. RNA-seq data for 112 normal and 1069 tumor specimens were downloaded from The Cancer Genome Atlas (TCGA) (www.cancer.gov/about-nci/organization/ccg/research/structural-genomics/tcga; accessed on 1 July 2021). Then, we calibrated and standardized the gene counts using the edgeR package (www.bioconductor.org/; accessed on 15 July 2021) version 3.34.0 in R language version 4.0.2 (cran.r-project.org/; accessed on 15 July 2021), and then heat maps of CD22 expression were constructed.

GEPIA (an online web server for large-scale expression profiling and interactive analysis) was used to analyze the levels of CD22 expression in breast cancer and normal tissues. GEPIA (gepia.cancer-pku.cn/; accessed on 1 March 2022) is a comprehensive and interactive web resource for analyzing cancer data and includes 9736 tumors and 8587 normal samples from both The Cancer Genome Atlas (TCGA) and Genotype-Tissue Expression (GTEx) [35].

CD22 expression in normal and invasive breast carcinoma tissues was analyzed using the online web tool TNM plot (tnmplot.com/analysis/; accessed on 1 March 2022). The plot includes RNA expression values for breast invasive carcinoma and normal samples. The TNM Plot database includes 56,938 unique multilevel quality-controlled samples: GeneChip from GEO: 3691 normal, 29,376 tumors, and 453 metastases, RNA-seq from GTEx: 11,215 normal, RNA-seq from TCGA: 730 normal, 9886 tumors, and 394 metastases, RNA-seq from TARGET: 12 normal, 1180 tumors, and 1 metastasis [36].

### 4.3. Cell Culture

Raji, K562, BT549, MCF7, MDA-MB-231, and mesenchymal stem cells (MSCs) cell lines were cultured in high glucose DMEM medium (SH30243.01, HyClone, Cytiva, South Logan, UT, USA) containing 10% fetal bovine serum (10099141C, Gibco, Australia) and antibiotics (Pen/Strep, 15140-122, Gibco, Waltham, MA, USA). When digesting cells, 0.25% trypsin-EDTA (25200072, Thermo Fisher Scientific, Waltham, MA, USA) was used.

### 4.4. Western Blot Analysis

Western blot analysis of breast cancer cell lines was performed in parallel with Raji cells (positive control for CD22), K562 cells (negative control for CD22), and MSCs (negative control for CD22). Protein concentrations were determined using a BCA assay (P0010, Beyotime, Shanghai, China). Lysates were separated by 7.5% (*w*/*v*) SDS-PAGE and transferred to polyvinylidene difluoride (PVDF) membranes, followed by incubation with the primary anti-CD22 polyclonal antibody (ab207727, EPR20061, Abcam, Cambridge, UK) at a 1:1000 dilution and an anti-rabbit conjugated secondary antibody (Rockland Immunochemicals, Gilbertsville, PA, USA). The signal was developed using an Odyssey Imaging System (ChemiDoc XRS+ system, Bio-Rad, Hercules, CA, USA). Furthermore, immunoblot analysis of these cell lines also revealed high specificity of Abcam anti-CD22 antibody as no multiple bands were observed.

### 4.5. Immunofluorescence

Cell lines were fixed with cold methanol for 30 min, washed with PBST (PBS, ZLI-9062, ZSGB-BIO, Beijing, China; TWEEN^®^ 20, V900548, Sigma-Aldrich, Darmstadt, Germany), incubated with the primary anti-CD22 (1:200, ab207727, Abcam, Cambridge, UK) antibody overnight at 4℃, and visualized using appropriate secondary fluorescent antibodies (ZF-0511, ZSGB-BIO, Beijing, China). Cells were subsequently counterstained with DAPI (diluted with water at a ratio of 3:7, C1006, Beyotime, Shanghai, China) and analyzed using a fluorescence microscope (Axio Observer A1, Zeiss, Oberkochen, Germany).

### 4.6. Immunohistochemical Staining of TNBC Specimens

Immunohistochemical (IHC) staining of 97 TNBC specimens (formalin-fixed and paraffin-embedded) was performed. Four micrometers thick TNBC and tonsil (positive control for CD22) sections were incubated in a citrate antigen retrieval solution in a pressure cooker. Then, slides were first washed with xylene (C_8_H_10_) and then washed with ethyl alcohol (CH_3_CH_2_OH). Then, to block endogenous peroxidases, hydrogen peroxide (H_2_O_2_) was used for ten minutes. Then, normal horse serum (1:10, ZLI-9024, ZSGB-BIO, Beijing, China) was used for blocking the slides. Then, the sections were incubated with primary anti-CD22 monoclonal antibody (1:100, ab207727, Abcam, Cambridge, UK) in PBS for 14 h at 4 °C. After the incubation of primary antibody, a two-step detection kit including secondary antibody (PV-9000, ZSGB-BIO, China) was used, and DAB was used for color development (ZLI-9018, ZSGB-BIO, Beijing, China) and then stained with hematoxylin (3201111, Wexis, Guangzhou, China).

### 4.7. Statistical Analysis

Clinical data were collected according to standardized protocols. To conduct statistical analysis, SPSS (Version 20, IBM, Armonk, NY, USA) software was used. Furthermore, the association between clinicopathological features and IHC staining was analyzed through chi-square and Fisher tests and shown in a table. *p* < 0.05 was considered to indicate statistical significance. Furthermore, correlation analysis between overall survival data of TNBC patients and CD22 expression of TNBC specimens was performed using MedCalc Statistical Software version 20.100 (MedCalc Software Ltd., Ostend, Belgium; https://www.medcalc.org; accessed on 1 September 2022). Additionally, a survival plot was constructed using GEPIA to show an association between different levels of CD22 expression and the overall survival of breast cancer patients.

## Figures and Tables

**Table 1:** Localization of CD22 in 97 TNBC Specimens.

Location of CD22 Expression	Number	Percent (%)
Membrane expression	23	23.71
Cytoplasm/Membrane expression	38	39.18
Negative	36	37.11

**Table 2:** Pathological scores for CD22 expression in TNBC specimens.

Pathological Score	Number of Specimens
Zero	36
One	13
Two	17
Three	31

**Table 3:** Analysis of the relationship between CD22 expression level and clinical pathological parameters in triple-negative breast cancer tissues (*n* = 97).

Clinical Parameters	Total	CD22-Negative (*n*, (%))	CD22-Positive (*n*, (%))	χ^2^	*p*-Value
Total number	97	36 (37.11)	61 (62.89)		
Age					
≤59	69 (71.13)	24 (24.74)	45 (46.39)	0.556	0.456
≥60	28 (28.87)	12 (12.37)	16 (16.50)		
Size of tumor					
pT1-pT2	80 (82.47)	26 (26.80)	54 (55.67)	4.163	0.041
pT3-pT4	17 (17.53)	10 (10.31)	7 (7.22)		
Lymph node status					
pN0	54 (55.67)	22 (22.68)	32 (32.99)	0.692	0.707
pN1-pN2	37 (38.14)	12 (12.37)	25 (25.77)		
pN3	6 (6.19)	2 (2.06)	4 (4.13)		
Histologic grade					
G1	9 (9.28)	2 (2.06)	7 (7.22)	1.740	0.419
G2	8 (8.25)	2 (2.06)	6 (6.19)		
G3	80 (82.47)	32 (32.99)	48 (49.48)		
Clinical stage					
I–II	76 (78.35)	26 (26.80)	50 (51.55)	1.267	0.260
III–IV	21 (21.65)	10 (10.31)	11 (11.34)		
Ki67 expression					
≤14%	4 (4.12)	1 (1.03)	3 (3.09)	0.000	1.000
>14%	93 (95.88)	35 (36.08)	58 (59.80)		

**Table 4:** Clinical trials of CAR-T cell immunotherapy for breast cancer.

Target	Phase	Status	Participants	Age (Years)	NCT Number
MSLN	I	Active, not Recruiting	186	>18	NCT02792114
EPCAM	I	Recruiting	30	18-65	NCT02915445
cMet	I	Completed	6	>18	NCT01837602
MUC1	I	Recruiting	69	>18	NCT04020575
HER2	I	Recruiting	220	>18	NCT04650451
CEA	I	Recruiting	75	18–80	NCT04348643
MUC1	I	Recruiting	112	>18	NCT04025216
ROR1	I	Recruiting	60	>18	NCT02706392
GD2	I	Recruiting	94	1–74	NCT03635632
CD44v6	I/II	Recruiting	100	0.5-75	NCT04427449

MSLN (Mesothelin); EPCAM (epithelial cell adhesion molecule); cMet (mesenchymal-epithelial transition factor gene); MUC1 (Mucin 1); HER2 (human epidermal growth factor receptor 2); CEA (carcinoembryonic antigen); ROR1 (receptor tyrosine kinase-like orphan receptor 1); GD2 (disialoganglioside); CD44v6 (CD44 variant domain 6).

## Data Availability

Not applicable.
